# The impact of agrochemical pollutant mixtures on the selection of insecticide resistance in the malaria vector *Anopheles gambiae*: insights from experimental evolution and transcriptomics

**DOI:** 10.1186/s12936-023-04791-0

**Published:** 2024-03-05

**Authors:** Christabelle G. Sadia, Jean-Marc Bonneville, Marius G. Zoh, Behi K. Fodjo, France-Paraudie A. Kouadio, Sebastien K. Oyou, Benjamin G. Koudou, Beatrice A. Adepo-Gourene, Stephane Reynaud, Jean-Philippe David, Chouaibou S. Mouahamadou

**Affiliations:** 1 University of Nangui Abrogoua, Abidjan, Côte d’Ivoire; 2 Centre Suisse de Recherches Scientifiques (CSRS), Abidjan, Côte d’Ivoire; 3 Laboratoire d’Ecologie Alpine (LECA) UMR 5553, Univ. Grenoble-Alpes, Univ. Savoie Mont Blanc, CNRS, 38000 Grenoble, France; 4 Vector Control Product Evaluation Centre (VCPEC)/Institut Pierre Richet, Bouaké, Côte d’Ivoire

**Keywords:** *Anopheles gambiae*, Agrochemical pesticides, Resistance selection, Metabolic resistance, Transcriptomics

## Abstract

**Background:**

There are several indications that pesticides used in agriculture contribute to the emergence and spread of resistance of mosquitoes to vector control insecticides. However, the impact of such an indirect selection pressure has rarely been quantified and the molecular mechanisms involved are still poorly characterized. In this context, experimental selection with different agrochemical mixtures was conducted in *Anopheles gambiae*. The multi-generational impact of agrochemicals on insecticide resistance was evaluated by phenotypic and molecular approaches.

**Methods:**

Mosquito larvae were selected for 30 generations with three different agrochemical mixtures containing (i) insecticides, (ii) non-insecticides compounds, and (iii) both insecticide and non-insecticide compounds. Every five generations, the resistance of adults to deltamethrin and bendiocarb was monitored using bioassays. The frequencies of the *kdr* (L995F) and *ace1* (G119S) target-site mutations were monitored every 10 generations. RNAseq was performed on all lines at generation 30 in order to identify gene transcription level variations and polymorphisms associated with each selection regime.

**Results:**

Larval selection with agrochemical mixtures did not affect bendiocarb resistance and did not select for *ace1* mutation. Contrastingly, an increased deltamethrin resistance was observed in the three selected lines. Such increased resistance was not majorly associated with the presence of *kdr* L995F mutation in selected lines. RNA-seq identified 63 candidate resistance genes over-transcribed in at least one selected line. These include genes coding for detoxification enzymes or cuticular proteins previously associated with insecticide resistance, and other genes potentially associated with chemical stress response. Combining an allele frequency filtering with a Bayesian FST-based genome scan allowed to identify genes under selection across multiple genomic loci, supporting a multigenic adaptive response to agrochemical mixtures.

**Conclusion:**

This study supports the role of agrochemical contaminants as a significant larval selection pressure favouring insecticide resistance in malaria vectors. Such selection pressures likely impact *kdr* mutations and detoxification enzymes, but also more generalist mechanisms such as cuticle resistance, which could potentially lead to cross-tolerance to unrelated insecticide compounds. Such indirect effect of global landscape pollution on mosquito resistance to public health insecticides deserves further attention since it can affect the nature and dynamics of resistance alleles circulating in malaria vectors and impact the efficacy of control vector strategies.

**Supplementary Information:**

The online version contains supplementary material available at 10.1186/s12936-023-04791-0.

## Background

Malaria is one of the deadliest mosquito-borne diseases in Africa mainly transmitted by *Anopheles* spp. [1, 2]. The recent World Health Organization (WHO) report shows 619,000 deaths due to malaria in Africa in 2021 [3]. Malaria control includes prevention through insecticide-based vector control in order to limit the transmission of pathogens [4]. Five classes of insecticides are used in malaria vector control but pyrethroids are the most widely used [4–7]. Their use relies essentially on two indoor methods: indoor residual spraying (IRS) and long-lasting insecticidal nets (ITNs). IRS and ITNs showed success between 2000 and 2015 by reducing the number of malaria cases in several countries [8]. Over the years however, the use of chemical insecticides has become less effective against malaria vectors, which now show strong resistance to most public health insecticides [9].

Insecticide resistance is widespread in insects [10]. Previous studies have shown that resistance in mosquitoes is mainly due to physiological adaptations, which include target-site mutations, increased insecticide metabolism or sequestration and altered insecticide penetration [11, 12]. Target site resistance is caused by non-synonymous mutations affecting the neuronal proteins targeted by insecticides [13, 14]. Such mutations are highly conserved in insects and well known in mosquitoes and their genotyping provides useful information to track resistance in the field [13–17]. Multiple target-site mutations conferring resistance to public health insecticides have been identified in African malaria vectors [18, 19]. The target protein of both pyrethroids and DDT is the voltage-gated sodium channel (VGSC) which is affected by knock-down resistance (*kdr*) mutations. A leucine–phenylalanine substitution at position 995 (L995F, orthologous to *Musca domestica Vgsc* codon 1014) [20], was first identified in West Africa and, therefore, named the ‘*kdr* West’ mutation [13]. A second mutation at the same codon (L995S), was also identified in East Africa. Nowadays, there is increasing evidence of the spread of the L995S mutation in West Africa and vice-versa [10, 21–24]. The targets of both organophosphate and carbamate insecticides are the acetylcholinesterases which include the *ace1* gene affected by the G119S resistance mutation in *Anopheles* spp. [25–28].

Beside target-site mutations, increased insecticide metabolism (*i.e.* metabolic resistance) has been reported in several African countries [29]. Such resistance phenotype is often caused by an increased activity of detoxification enzymes including cytochrome P450 monooxygenases, carboxylesterases, glutathione *S*-transferases, and UDP glycosyltransferases, though other protein families can also be involved [11]. Such increased activity is often caused by gene over-expression though structural modifications contributing to enhanced insecticide metabolism populations have also been identified in malaria vectors [29]. Given the high diversity and functional redundancy of insect detoxification enzymes, the identification of those conferring resistance to insecticides proved to be challenging [30]. Although target-site mutations and metabolic resistance clearly play a major role in conferring resistance to public health insecticides, other physiological changes such as altered insecticide penetration caused by cuticle thickening or structural modification have also been shown to contribute to resistance [31]. Indeed cuticular proteins like CPAP3E, CPLCG4 and CPLCG5 have been frequently associated with mosquito resistance through cuticle thickening [32–34]. Genes coding for P450 cytochromes of the Cyp6M and Cyp6Z families, together with GSTE2, are involved in mosquito resistance to insecticides [35–37].

Though the mass distribution of LLINs and IRS acted as a major selection pressure leading to pyrethroid resistance in Africa [38, 39], the intensive use of the same insecticide families for crop protection also represents a significant selection pressure undergone by *Anopheles* populations located in agricultural areas [40]. Indeed, mosquito larvae found in these ecosystems are exposed to a wide range of pesticides used against crop pests while adults may also be impacted by agricultural spraying operations [41, 42]. In addition, non-insecticide molecules such as herbicides and fungicides can also have adverse effects on mosquitoes and may contribute to resistance selection through chemical stress response mechanisms [43]. Overall, an increasing number of studies support the role of agricultural xenobiotics as a key selection pressure contributing to insecticide resistance in mosquitoes, though the underlying mechanisms have not been fully characterised [44, 45].

In this context, the aim of this work was to explore the potential of agrochemical mixtures present in *Anopheles gambiae* breeding sites to select for inherited resistance to vector control insecticides at the adult stage. A field derived *An. gambiae* line was experimentally selected at the larval stage for 30 generations with three different mixtures containing agrochemicals commonly used in agriculture in Africa: (i) a mixture of insecticide-based formulations, (ii) a mixture of fungicide and herbicide formulations, and (iii) a mixture containing both insecticide and non-insecticide formulations. Comparative bioassays with the pyrethroid deltamethrin and the carbamate bendiocarb were used to monitor the resistance of adults to vector control insecticides across generations. The impact of each selection regime on resistance mechanisms was investigated by genotyping target-site mutations and whole transcriptome analysis. Results are discussed in regards to the impact of agriculture on the management of insecticide resistance in malaria vectors.

## Methods

### Mosquitoes

A field-derived colony of *An. gambiae* (form S) from Tiassalé in southern Côte d'Ivoire was used as the parental strain in the present study. This colony, hereafter Tiassale-S, has been maintained since 2015 at the Centre Suisse de Recherches Scientifiques (CSRS) in Côte d'Ivoire without selection and now exhibits a phenotype of low resistance to public health insecticides compared to contempory field mosquitoes from the locality of Tiassalé. The mosquitoes were reared under standard tropical rearing conditions (27 ± 3 °C and 75 ± 10% humidity under 12:12 photoperiod). Larvae were fed on cat food and adults on 5% honey solution. Adult females were blood fed membrane feeding (Hemotek).

### Agrochemical mixtures

Seven commercial formulations of products frequently used in agriculture in Côte d’Ivoire (each containing single or multiple insecticides, herbicides and fungicides and their adjuvants) were chosen according to surveys of farmers in Côte d’Ivoire to identify the pesticides commonly used by farmers [40]. These formulated products were combined to constitute the three agrochemical mixtures used for selection (Table 1). The stock insecticide mixture solution contained 3.3 ml/L Legumax® (deltamethrin); 2.7 ml/L K-Optimal® (lambda cyhalothrin, acetamiprid); 1.5 ml/L Verso 480® (chlopyriphos ethyl) and 66.7 g/L Furadan® (carbamate). The stock non-insecticide mixture contained 5.3 ml/L Banko plus® (fungicides, chlorothalonil and carbendazim); 5.3 ml/L Glyphader® (herbicide, glyphosate); 5.3 ml/L Garil® (herbicide, amide pyrimidine). A stock solution mixing all insecticide and non-insecticide compounds was also prepared (Table 1).

**Table 1:** Composition of agrochemical mixtures used for larval selection

Mixtures	Trade name	Active ingredient (AI)	Chemical class	AI Concentration	Application dose (solvant)
Insecticides (Ins line)	Furadan®	Carbofuran	Carbamates	50 g/Kg	66.7 g/1 L (water)
	K-optimal®	Acetamipride Lambda-cyhalothrin	Neonicotinoids Pyrethroids	15 g/L 20 g/L	2.7 mL/1 L (water)
	Legumax®	Deltamethrin	Pyrethroids	12 g/L	3.3 mL/1 L (water)
	Verso 480®	Chlopyrifos ethyl	Organophosphates	480 g/L	1.5 mL/1 L (water)
Others (non-ins line)	Banko Plus® (fungicide)	Chlorothalonil Carbendazine	Organochlorines Carbamates	550 g/L 100 g/L	5.3 mL/1L (water)
	Garil® (herbicide)	Propanil Trichlopyr	Amides Pyridines	360 g/L 72 g/L	5.3 mL/1L (water)
	Glyphader® (herbicide)	Glyphosate	Amino-phosphonates	360 g/L	5.3 mL/1L (water)

### Controlled selection

The parental line was divided into four distinct lines, each subjected to a different selection regime. The Control line (Cntrl line) was maintained without selection pressure and served as control in all experiments. The Insecticide line (Ins line) was produced by exposing L2 larvae to a mixture of five insecticides from the pyrethroid, neonicotinoid, carbamate and organophosphate chemical families (Table 1). The Non-insecticide line (Non-ins line) was produced by exposing L2 larvae to a mixture of five non-insecticide molecules, two fungicides from the organochlorine and carbamate families and three herbicides from the amide, pyridine and aminophosphonate families. The mixture line (mix line) was selected using the mixture of Insecticide and Non-insecticide formulations*.* For each line, selection for resistance in mosquitoes was performed by exposing stage II larvae (~ 300) in tanks containing 300 mL of water and agrochemical mixtures for 24 h to the different agrochemical mixtures. Surviving larvae were rinsed and transferred to clean tap water and reared. As soon as they emerged, the adults of both sexes, which were placed in wire cages, were allowed to mate freely and membrane feeding (Hemotek) was used to generate the eggs of the next generation. Selection was carried out over 30 successive generations using a dilution killing 20% of the larvae (LD_20_) in the different lines (see Table 2). The selection pressure was maintained around the LD_20_ throughout the selection process by adjusting the doses every five generations. The new LD_20s_ were determined using PoloPlus software.

**Table 2:** Concentrations of agrochemical mixtures used for larval selection

	Insecticides line (LD_20_)	Non-insecticides line (LD_20_)	Mixture line (LD_20_)
G0–G5	5 µL/100 mL (water)	500 µL/100 mL (water)	11 µL/100 mL (water)
G5–G10	8 µL/100 mL (water)	900 µL/100 mL (water)	20 µL/100 mL (water)
G10–G15	12 µL/100 mL (water)	1200 µL/100 mL (water)	25 µL/100 mL (water)
G15–G20	15 µL/100 mL (water)	1300 µL/100 mL (water)	32 µL/100 mL (water)
G20–G25	25 µL/100 mL (water)	1330 µL/100 mL (water)	40 µL/100 mL (water)
G25–G30	47 µL/100 mL (water)	1350 µL/100 mL (water)	46 µL/100 mL (water)

### Insecticide resistance monitoring

The resistance level of each line to bendiocarb and deltamethrin, two insecticides used in vector control, was monitored at the adult stage every five generations. Bioassays were carried out according using test tubes with filter papers impregnated with either 0.1% bendiocarb or 0.05% deltamethrin [46]. At least four batches of 20 to 25 non-blood fed 2–5 days old females were used. Mortality was recorded after one hour of insecticide exposure and a 24 h recovery time during which the mosquitoes were provided a 5% honey solution. The Abbot formula correction was applied when the control mortality rate was between 5 and 20% and assays were discarded if mortality in control exceeded 20% [47]. The mortality of each selected line to each insecticide was compared to that of the unselected line at the same generation using a Fisher test (N ≥ 4).

### Target-site mutations

The frequencies of the *kdr* West L995F and *ace1* G119S target-site mutations were monitored in each line by individual genotyping at generations G0, G10, G20 and G30. Genomic DNA was extracted using 2% CTAB as previously described [48]. *Kdr* L995F and *ace1* G119S mutations were genotyped using the allele-specific TaqMan qPCR methods as described. Each reaction mixture contained 5 μL of 2X sensimix (Bio Rad), 3.875 μL of nuclease-free water, 0.125 μL of TaqMan probes and 1 μL of gDNA. Quantitative PCR reactions were performed on a CFX 96 Real Time system (Bio-Rad technologies, California, USA) with the following amplification conditions: 95 °C for 10 min, followed by 40 cycles of 95 °C for 10 s and 60 °C for 45 s. For each mutation, individuals were scored as homozygous susceptible/resistant or heterozygous based on the intensity of the HEX/FAM channels at the end of the PCR reaction as compared to positive and negative samples of known genotypes. For each generation, the *kdr* and *ace1* genotype frequencies were compared between selected and unselected lines using Genepop sofware 4.0.10 based on a chi^2^ test.

### RNA-seq library preparation and sequencing

The transcriptome of each selected line was compared to the control line using RNA-seq at generation G30. For each line, four pools of 30 three-day-old non-blood fed females (not exposed to insecticide) were collected and stored in RNA-later at − 20 °C. Total RNA was extracted from each pool using Trizol (Life Technologies) according to manufacturer’s instructions, and treated with DNase to remove genomic DNA contaminants. RNA-seq libraries were prepared from 150 ng total RNA using NEBNext® Ultra™ II Directional RNA library Prep Kit for Illumina (New England Biolabs) following manufacturer’s instructions. Libraries were quantified using the Qubit DNA BR assay (Thermofisher Scientific) and quality checked using Bioanalyzer DNA 1000 assay (Agilent). Libraries were sequenced in multiplex as single 75 bp reads on a NextSeq 500 sequencer (Illumina) by Helixio (Clermont-Ferrand, France). After demultiplexing and quality check using FastQC, reads were loaded into Strand NGS V 3.2 (Strand Life Sciences) and mapped against the AgamP4 assembly and AgamP4.12 geneset using the following parameters: min identity 90%, max gaps 5%, min aligned length 35 bp, ignore reads with more than 5 matches, trim 3’ ends of reads with average quality < 20, Kmer size 11, match score 1, mismatch score 4, gap opening penalty 6, gap extension penalty 1. Mapped reads were then filtered based on their sequence quality and mapping quality as follows: Mean read quality $\ge$ 20, number of N ≤ 5, alignment score $\ge$ 90, mapping quality $\ge$ 120, number of match = 1. The remaining reads (~ 90% of sequenced reads) were used for subsequent analyses.

### Differential gene transcription

Differential transcription analysis was performed on Strand NGS V3.2. This analysis was performed on all protein coding genes with normalisation and quantification being performed according to the DESeq algorithm [49]. Only the 10357 genes showing a coverage ≥ 4 reads/kb in all replicates across all conditions were kept for further analysis. Transcription levels between each selected line and the control line were then compared across the four biological replicates using an ANOVA followed by a Tukey HSD test. P values were adjusted for multiple testing corrections using the false discovery rate method [50]. Genes showing a transcription ratio ≥ 1.5 fold in either direction and a P value ≤ 0.005 in any selected line as compared to the parental line were considered differentially transcribed following selection.

For each selected line, genes significantly over- and under-transcribed as compared to the control line were subjected to a Gene Ontology (GO) term enrichment analysis using the functional annotation tool DAVID [51]. Reference gene list was constituted by the 10357 genes detected by RNA-seq and test lists were constituted from over- and under-transcribed genes in each line. GO term frequencies in selected vs unselected line were compared in a Fisher’s Exact test, and terms with a P value < 0.05 upon FDR multiple testing correction were kept [50]. A panel of 193 genes were selected from Agam P4.12 geneset as candidates possibly contributing to xenobiotic resistance. These genes included known insecticide targets, detoxification enzymes (cytochrome P450s, carboxylesterases and transferases), ABC-transporters, cuticle proteins, enzymes associated with redox stress, nervous receptors and putative insecticide binding proteins (see Additional file 4: Table S1). Heat maps reflecting transcription profiles of differentially expressed resistance candidate genes across all lines were generated using TM4 Multi-experiment Viewer (MeV) software [52].

### Sequence polymorphism

Small Nucleotide Polymorphisms (SNPs) were called from transcriptomic sequence data using Strand NGS V 3.2 against all protein-coding genes of the AgamP4.12 geneset using standard parameters (ignore homopolymer stretches greater than 4 bp and adjacent positions, coverage ≥ 30 and ≤ 5000, reads supporting the variant allele ≥ 2, base quality ≥ 20, variant confidence score ≥ 200 and strand bias ≤ 25). Among the variations called, only substitutions and indels were retained for further analyses. A principal component analysis [53] was then used to visualise the genetic divergence of each line to the AgamP4 reference genome. PCA was performed on the frequency of all bi-allelic variations identified in each replicate of all lines using the Ade4 R package [54]. Genic effects were then computed based on the longest transcript for each gene according to the AgamP4.12 geneset and sorted as affecting (non-synonymous) or not (synonymous) the protein sequence. Selection signatures were investigated using the bi-allelic SNPs that were polymorphic (i.e. showing a > 5% allele frequency variation between the control parental line and at least one selected line) in two ways. First, a selection was made of differential SNPs, i.e. those showing a clear difference in frequency between the selected and non-selected lines. For this purpose, the mean variant frequencies were compared in a Student's T test, and SNPs with a P-value < 0.0005 after FDR correction were retained. This stringent P-value threshold still retained 2.5 to 4.8% of the SNPs, depending on the selected line. A SNP score was then computed for each differential SNP based on its absolute frequency variation between the selected line and the control line. The score of non-differential SNPs was set to 0 while differential SNP scores were calculated as follows: Score = Abs[(%freq_Selected_) − (%freq_control_)]/50, where ‘%freq’ is the frequency in % of the variant allele. In this way, an allele showing a 50% frequency variation in a selected line scores 1, and an allele absent in the control line and fixed in a selected line scores 2. A gene Diff score was finally computed by summing SNP scores and dividing by the number of polymorphic SNPs in each gene. A second approach consisted in assessing F_ST_ departure from neutrality using the Bayesian method implemented in BayeScan version 2.1 [55]. A separated analysis was performed consisting in contrasting the selected line versus the control line across their four replicates. Default settings were used except that prior odd was set to 1000 in order to increase stringency. SNPs showing a Bayescan Q‐value of zero were considered as ‘Outliers’. Outliers represented 2.9 to 4.1% of all SNPs, depending on the selected line, and were counted per gene. The percentage of outlier SNPs in each gene was then plotted along chromosomes.

## Results

### Insecticide resistance dynamics during the selection process

**Fig. 1:**
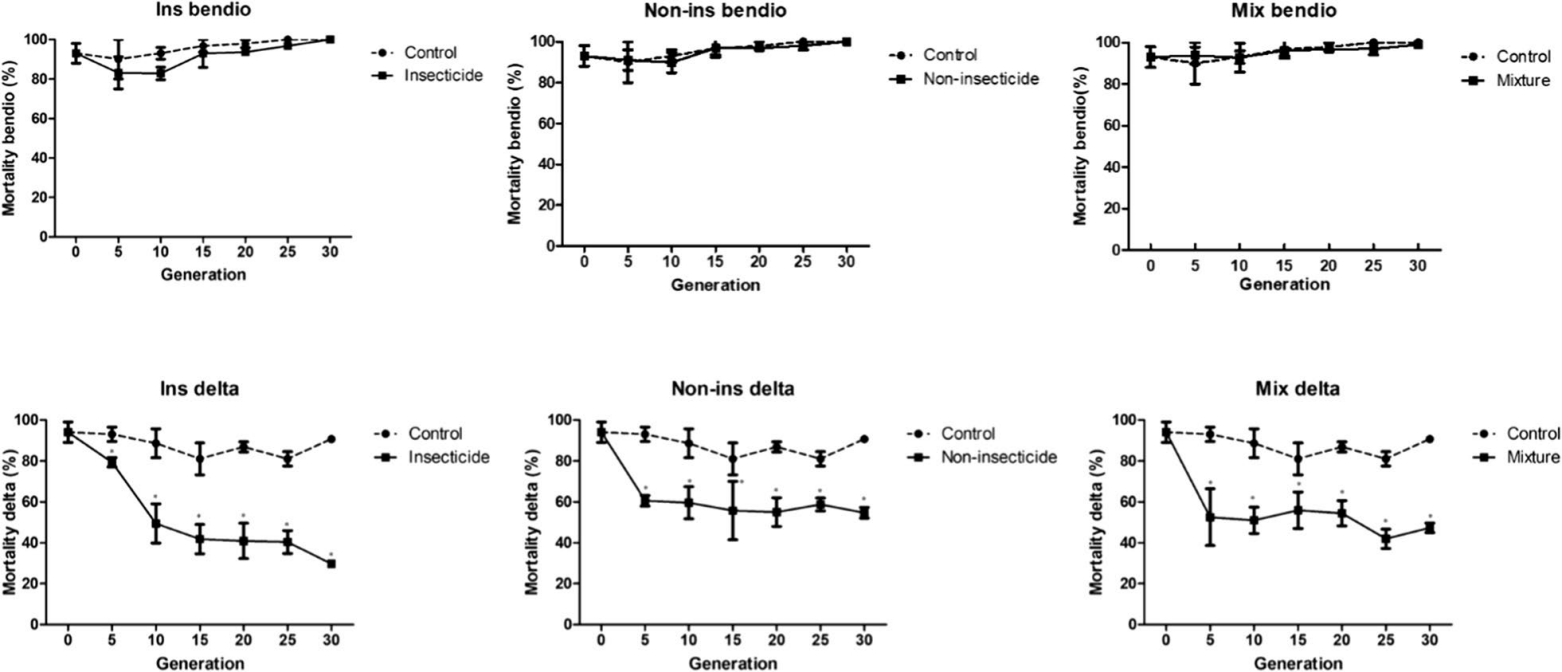
Evolution of adult resistance to bendiocarb and deltamethrin in each selected line compared to the control line. For each line, insecticide resistance levels are shown as % mortality to 0.1% bendiocarb and to 0.05% deltamethrin. * Indicate significantly distinct mortalities using Fisher’s exact test (P < 0.05), and error bars show SD in means 80 < n > 100 computed from each replica tube

The resistance of adults to deltamethrin and bendiocarb was monitored in each line during the selection process (Fig. 1). High mortality rates to deltamethrin (94.1%) and to bendiocarb (92.7%) were obtained with the parental line at G0, supporting the low frequency of resistance alleles at the beginning of the selection process. Larval selection by the agrochemical mixtures had no impact on bendiocarb resistance, despite the presence of carbamates and organophosphates (both targeting the acetylcholinesterase) in the agricultural insecticide mixture. Conversely, an increased deltamethrin resistance was observed in response to larval selection with agrochemical mixtures. This increased resistance was significant in all selected lines from G5 onwards and continued to increase up to G30. The highest resistance level was reached in the ins line selected with the insecticide mixture (29.8% mortality at G30) while the two other non-ins and mix lines were less resistant at G30 (non-ins line 54.74% and mix line 47.36% mortality at G30).

### Target-site mutations

**Fig. 2:**
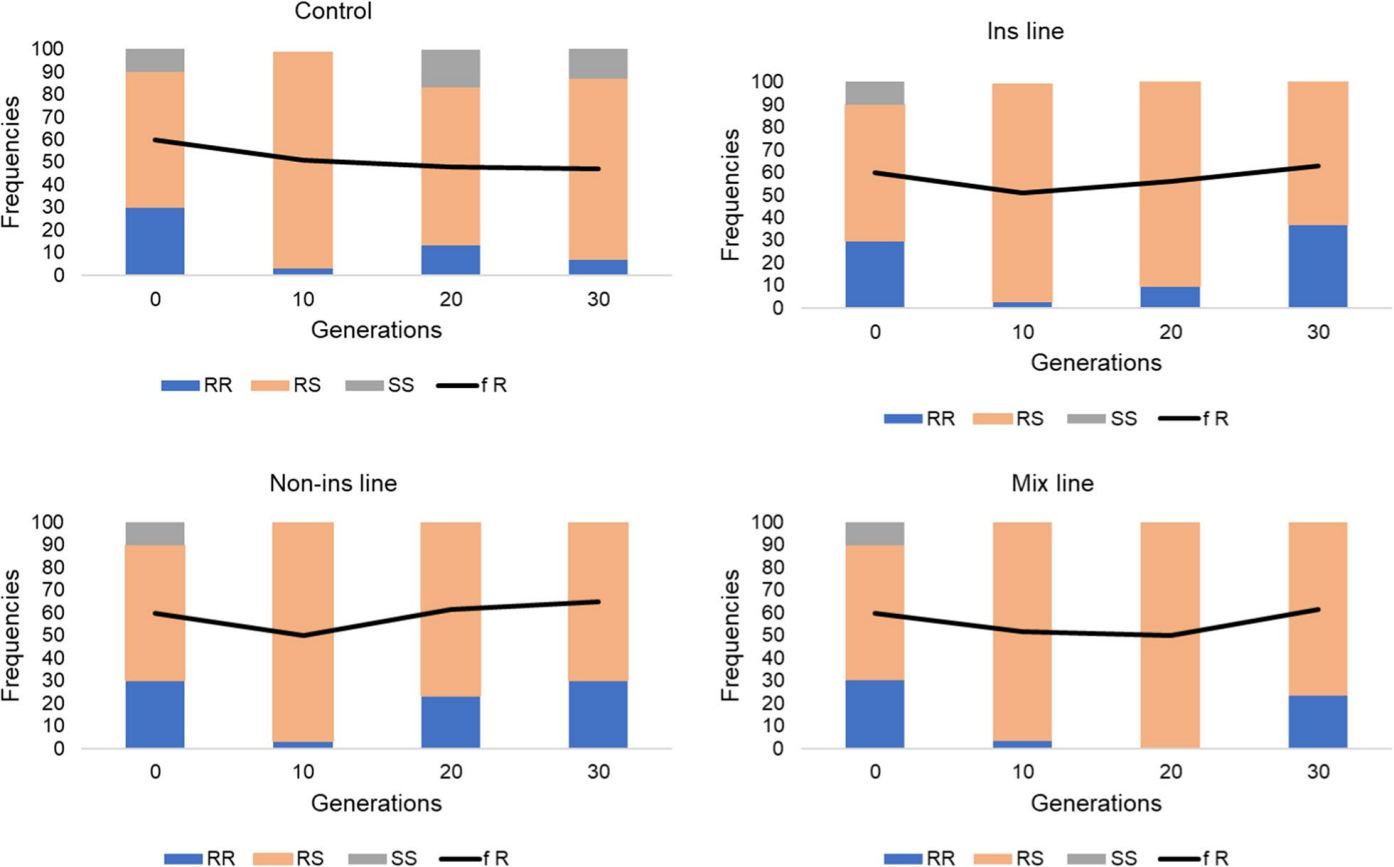
Evolution of the *kdr* L995F mutation frequency during the selection process. Coloured bars show the genotype frequencies, as assayed from 30 individuals. Generation 0 corresponds to the parental line. Blue: 995FF kdr (resistant) homozygotes; Orange: 995FL heterozygotes; Grey: 995LL (wildtype) homozygotes. Black line: 995F allele frequency

**Fig. 3:**
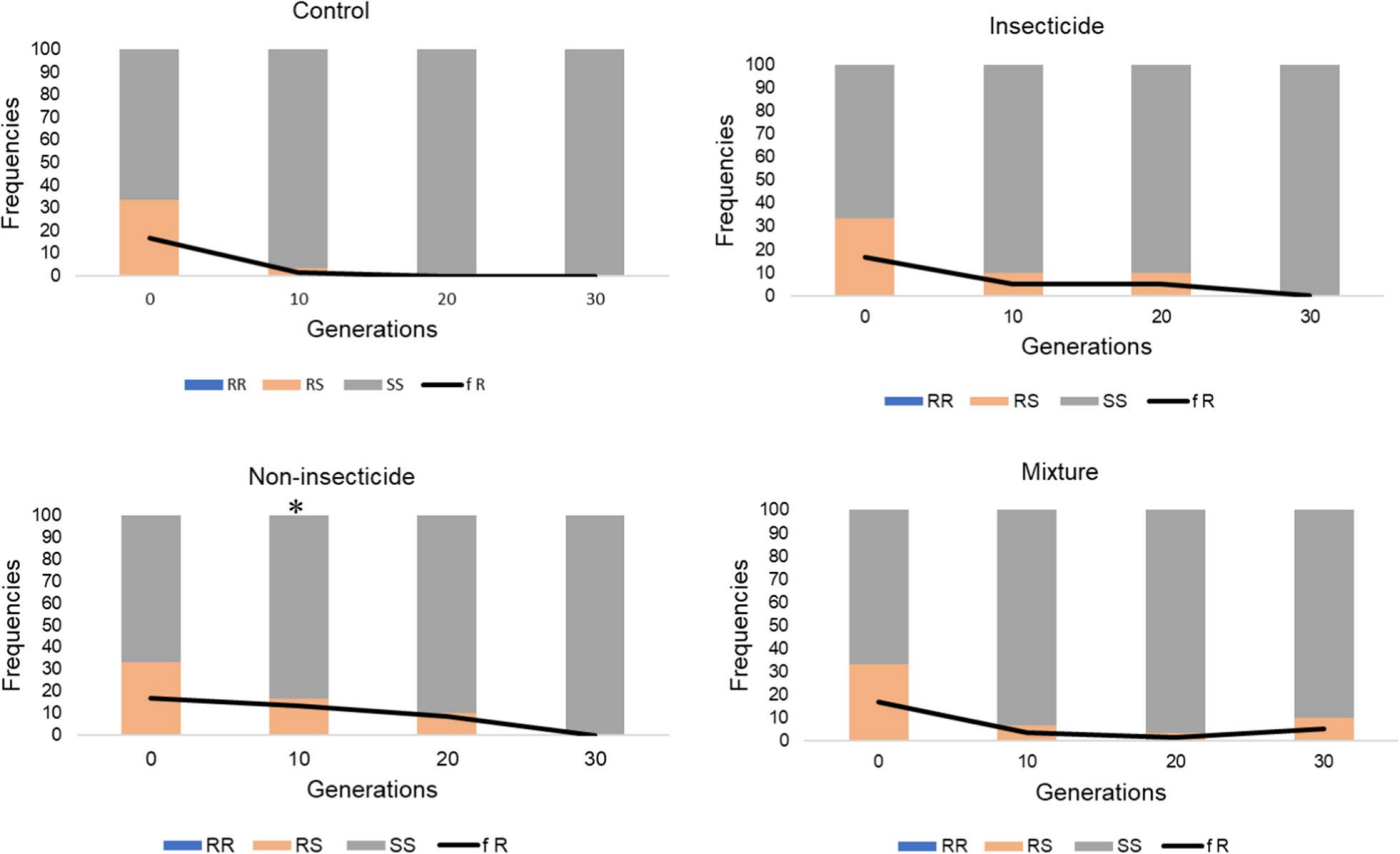
Evolution of the Ace1 mutation frequency during the selection process. Coloured bars show the genotype frequencies, as assayed from 30 individuals. Generation 0 corresponds to the parental line. Blue: 119SS (resistant) homozygotes; Orange: 119GS heterozygotes; Grey: 119GG (wildtype) homozygotes. Black line: 119S allele frequency

The evolution of *kdr* mutations affecting the voltage-gated sodium channel and conferring resistance to pyrethroids and DDT was monitored through the selection process. The *kdr* East (L995S) mutation was not detected in the parental line and thus not further quantified during selection. The *kdr* mutation L995F was present in the parental line at a frequency of 60% at G0 (Fig. 2). Its frequency significantly decreased to reach 47% in the non-selected line at G30. Conversely, the frequency of the *kdr* L995F mutation remained stable in all selected lines (G30 frequencies of 63%, 65% and 62% in the Ins, Non-ins and Mix lines respectively). Genotypes carrying the *kdr* L995F mutation were significantly more represented in all selected lines as compared to the control line at G30. The *ace1* mutation G119S affecting the acetycholinesterase and conferring resistance to carbamates and organophosphates was present in the parental line at a low initial frequency (17%). Its frequency gradually decreased through generations in all lines selected or not with agrochemical mixtures (Fig. 3). At G30, the final *ace1* mutation frequencies were 0%, 0%, 5% and 0% in the ins, non-ins, mix and control lines respectively. However, a significant difference of genotype frequency was found between the control line and the non-ins line at G10 (chi^2^ test P < 0.05).

### Differential gene expression

Among the 10357 genes detected by RNA-seq, 775 were considered as significantly differentially transcribed in at least one selected line as compared to the control line (≥ 1.5 fold-change in either direction and corrected P value ≤ 0.005, Additional file 4: Table S1). A total of 472 genes were over-transcribed in at least one selected line with 294, 299 and 216 identified in the Ins, Non-ins and Mix lines respectively (Additional file 1: Figure S1). Among them, 111 genes were shared by two selected lines and 113 genes were shared by the three selected lines. Only 304 genes were under-transcribed in at least one selected line with 182, 156 and 177 identified in the Ins, Non-ins and Mix lines, respectively. Among them, 83 genes were shared by two selected lines and 64 genes were shared by the three selected lines.

Gene ontology enrichment analyses identified biological processes enriched from genes significantly over- and under-transcribed in each selected line (Additional file 2: Figure S2). Only a few GO terms were found significantly enriched from under-transcribed genes with a large overlap across the three selected lines. These included two terms associated with endopeptidase activity enriched in all lines and the GO term ‘hydrolase activity’ identified in both the Ins and the Mix lines. Multiple GO terms were found significantly enriched from over-transcribed genes, with again a good overlap across the three selected lines. These essentially included terms associated with P450 activity and detoxification (‘oxidoreductase activity’; ‘monooxygenase activity’; ‘iron ion binding’; ‘haem binding’; ‘flavin adenine dinucleotide binding’) together with terms associated with the insect cuticle (‘structural constituent of cuticle’;’chitinase activity’; ‘chitin binding’).

**Fig. 4:**
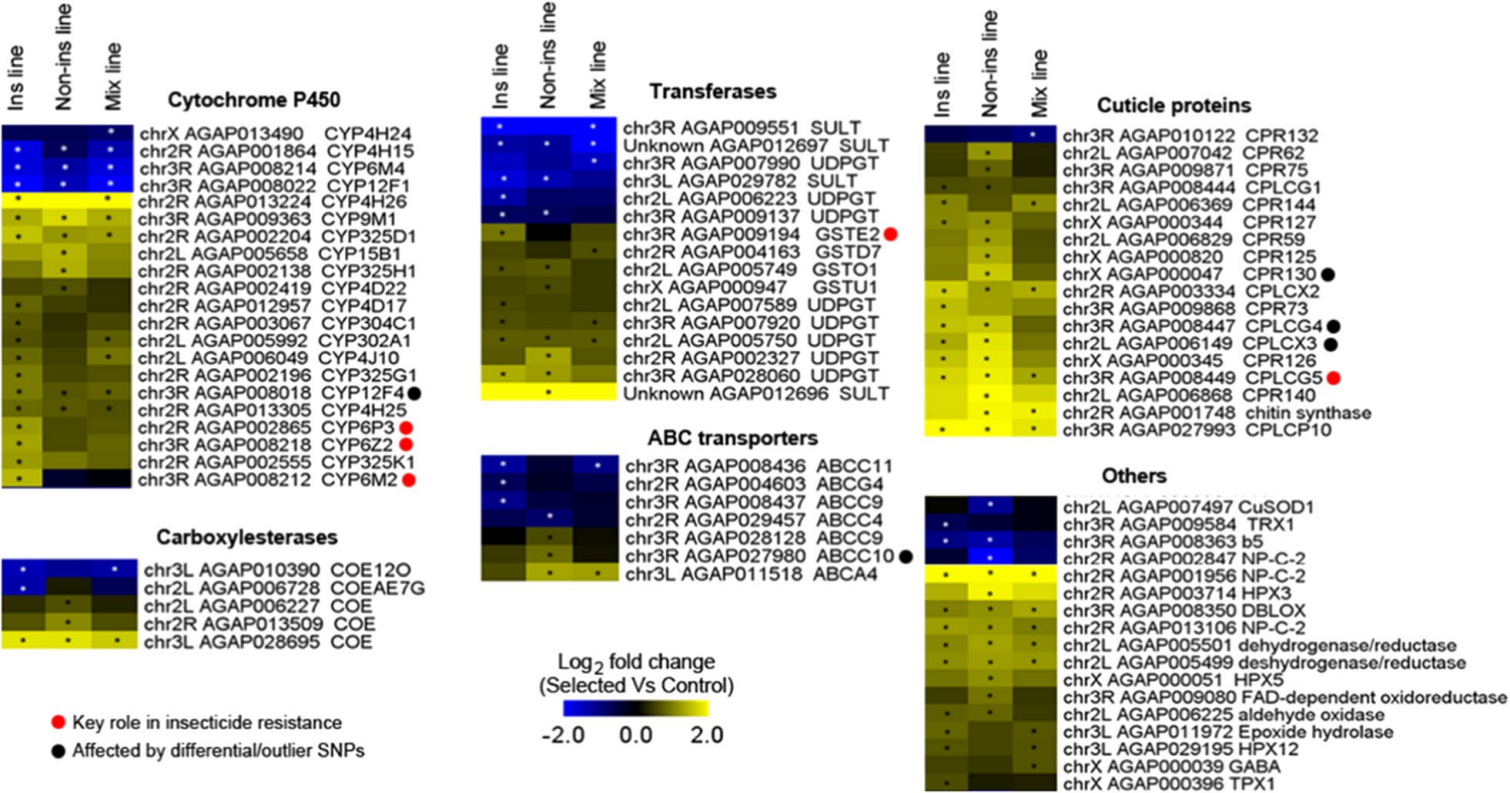
Expression profiles of candidate resistance genes in each selected line. Gene transcription levels were quantified by RNA-seq after 30 generations of selection. Only genes showing a significant differential transcription level between at least one selected line and the control line are shown (*: FC ≥ 1.5-fold in either direction and corrected P value ≤ 0.005). Red dots indicate genes known as contributing to insecticide resistance in malaria vectors. Black dots indicate genes affected by differential or outlier SNPs

A total of 63 candidate genes potentially involved in insecticide resistance were over-transcribed in at least one selected line while only 23 candidate genes were found under-transcribed (Additional file 1: Figure S1). Among over-transcribed candidate genes, half were over-transcribed in at least two selected lines, including 14 over-transcribed in the three selected lines. Over-transcribed candidate genes include 17 P450s, 3 carboxylesterases, 10 transferases, 3 ABC transporters, 17 cuticle proteins and 13 other candidates (Fig. 4). Most P450s showed an over-transcription in the Ins line with three of them (*CYP6P3*, *CYP6M2* and *CYP6Z2*) being known as able to metabolise insecticides [37, 56, 57]. Four P450s (*CYP9M1*, *CYP325D1*, *CYP12F4* and *CYP4H25*) were over-transcribed in the three selected lines, with *CYP12F4* also showing a significant selection signature (see below). Other over-transcribed detoxification genes include four GSTs, five UDPGTs, one sulfotransferase, three carboxylesterases, three ABC transporters and other enzymes including an aldehyde oxidase and an epoxide hydrolase. *GSTE2*, known as able to metabolise DDT, was over-transcribed in the Ins line. Most of the 17 over-transcribed cuticle proteins were identified in multiple selected lines with CPLCX2, CPLCG5 and CPLPCP10 over-transcribed in all selected lines, and CPLCG5 previously shown to play a key role in cuticle resistance [34]. Three cuticle proteins (CPR130, CPLCG4 and CPLCX3) were also associated with genomic selection signatures (see below). Among genes likely involved in response to oxidative stress, three haem peroxidase (HPX3, HPX5 and HPX12) and one thioredoxin peroxidase were over-transcribed in one or multiple selected lines. Finally, two cholesterol-like transporters (Niemman-Pick type C2 proteins, NPC2) potentially capable of binding xenobiotics were over-transcribed in all selected lines.

### Polymorphism variations

More than 60 K SNPs were detected across all lines. When projected by Principal Component Analysis (PCA), the three first axes accounted for 96.3% of the total variance (Additional file 3: Figure S3). This included > 80% for the first axis which did not separate the selected and unselected lines and rather reflected the polymorphism between the parental line and the reference genome. The second axis (12.5%) opposed the control line to all selected lines in a balanced manner, supporting a common adaptive response to xenobiotics. Finally, the third axis (2.6%) opposed the Ins line and the Non-Ins line, implying specific components of adaptive response of lesser magnitude; the Mix line was located in-between, supporting its intermediate adaptive response.

**Fig. 5:**
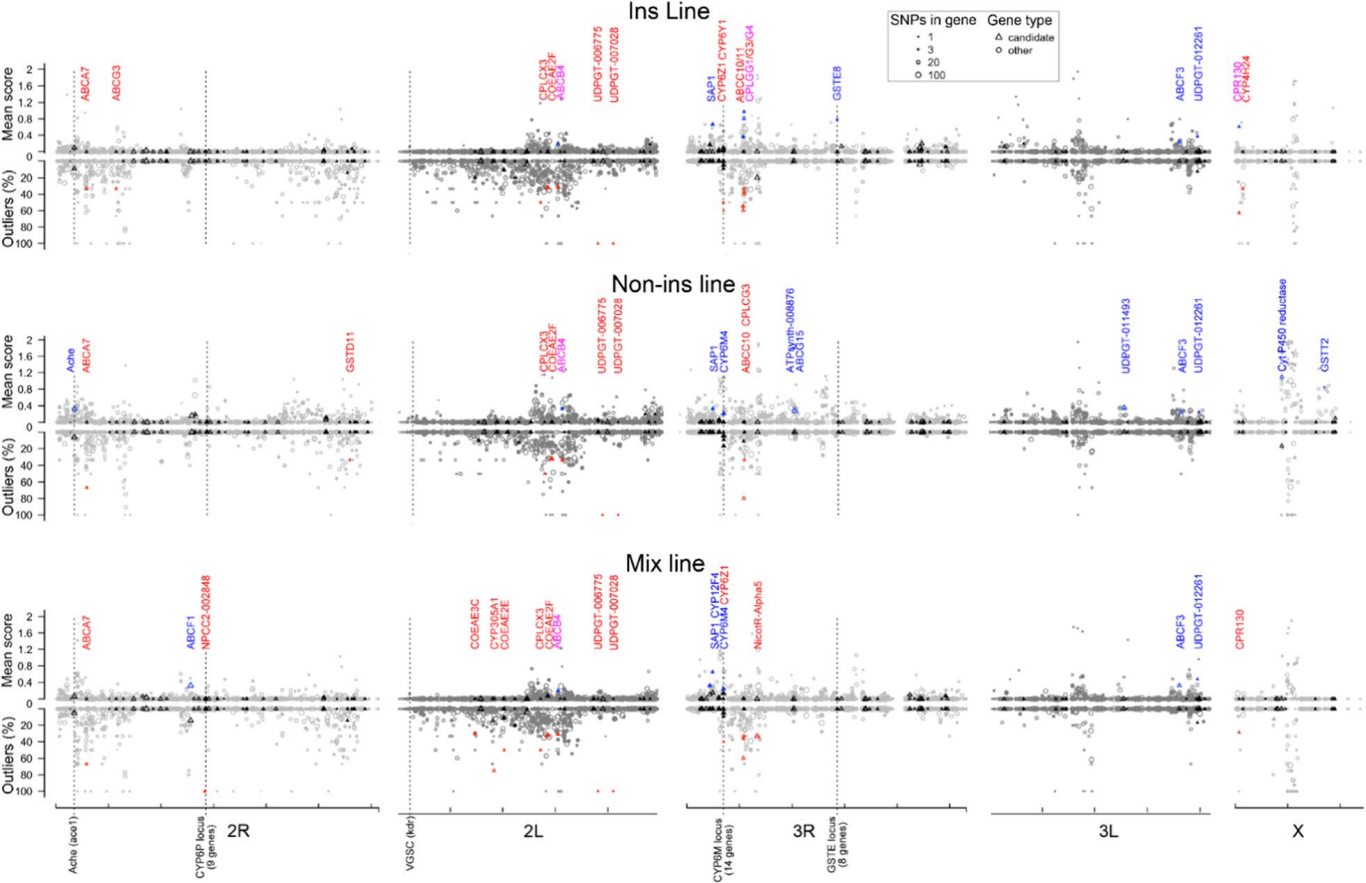
Selection signatures observed in each selected line. SNPs diverging between the control line and each selected line were identified using a frequency-based approach (Diff SNPs) and a FST-based approach (outlier SNPs) and then averaged by gene (see methods). The upper Y axis shows the mean Diff SNP score per gene. The lower Y axis shows the proportion of outliers per gene. Symbol size increases with the number of polymorphic SNPs per gene. Triangles and circles denote candidate and non-candidate genes, respectively. Filled symbols indicate the presence of at least one differential or outlier SNP affecting the protein sequence. Blue and red symbols indicate candidate genes with a mean differential score > 0.4 or > 20% outliers, respectively; the corresponding gene names are indicated. Loci commonly associated with insecticide resistance in *An. gambiae* are indicated by dashed lines. The genomic scale shows chromosome arms with ticks every 10 Mb

The 50 K SNPs that were polymorphic between the control line and at least one selected line were used (Additional file 5: Table S2). As expected from transcriptomic data, most of these SNPs fell (98%) within gene boundaries, covering 4406 genes (i.e. 42% of all RNA detected genes). Differential SNPs (Diff SNPs) were defined as those whose frequency varied significantly between any selected line and the control line (see “Methods”). These include 3744, 4280 and 3862 Diff SNPs for the Ins, Non-ins and Mix lines, respectively. Summing up the Diff SNP scores supported a highest genetic divergence from the control line for the Ins line (total weight ~ 100), followed by the Mix line (total weight ~ 90) and the Non-ins line (total weight ~ 70). The Fst-based approach identified 2179, 1554 and 1994 Outlier SNPs, in the Ins, Non-ins and Mix lines, respectively, supporting the same divergence ranking between the selected lines as compared to the control line. Diff SNP and Outlier SNP densities often coincided between the two approaches, revealing multiple regions potentially under selection (Fig. 5). These regions often coincided across selected lines though no decreased genetic diversity was observed in the control line (53 K SNPs detected in the control line versus 45 K to 47 K in the selected lines), rather supporting a common multi-genic adaptive response to chemical stress than drift in the control line only during insectarium rearing. Candidates genes affected by Diff/Outlier SNPs in regions showing shared selection signatures included three transporters (ABCB7, ABCB4 and ABCF3), three UDPGTs (*AGAP006775 AGAP007028* and *AGAP012261*), two cuticular proteins (CPLCX3 and CPLCG3) and the sensory appendage protein SAP1. Other candidate genes were found in regions showing more specific selection signals such as *GSTE8* (Ins line only, Chr 3R), CPR130 (Ins and Mix lines, Chr X) and *ace1* (Non-ins line only, Chr 2R). Among the 14 P450s of the *CYP6M* cluster on Chr 3R (which includes P450s known to metabolise insecticides and other xenobiotics) [56, 58], three of them were affected in the Ins or the Mix lines (*CYP6Y1, CYP6M4* and *CYP6Z1*) but not in the Non-ins line. Another interesting signal was observed on Chr 3R in a gene cluster containing 28 cuticle proteins (which includes CPLCG5 known to contribute to pyrethroid resistance) and from which the neighbouring genes CPLCG3 and CPLCG4 were identified in multiple selected lines. Finally, no selection signature was observed in the vicinity of the VGSC gene *AGAP004707* (containing *kdr* mutations), but its low expression level prevented the detection of polymorphic SNPs in this region.

## Discussion

The role of agriculture in the development of insecticide resistance in mosquitoes is increasingly being recognized. Indeed, laboratory work has shown the potential of agrochemicals to induce or select for an overexpression of resistance genes in both larvae and adults [44, 59]. In the field, susceptibility testing of adult mosquitoes in areas of high agricultural activity often revealed an increased resistance associated with the expression of detoxification enzymes [19, 42]. This is because some mosquito larvae developing in these ecosystems are subject to selection pressure as well as adult mosquitoes present during agricultural spraying campaigns [60]. The long term impact of agriculture on the selection of resistance in mosquitoes is also supported by the fact that insecticides used in agriculture are often similar (same families and modes of action or same molecules) as those used in public health [40]. Although non-insecticidal agrochemicals were also shown to affect mosquito tolerance to insecticides [61, 62], the effect of complex agrochemical mixtures has less been studied. In this context, the present study aimed at combining controlled selection and molecular approaches to study the impact of larval selection by insecticide and non-insecticide agrochemical mixtures on the selection of insecticide resistance mechanisms in *An. gambiae*.

### Larval selection with agrochemical mixtures select for increased resistance in adults

The present study confirmed that larval selection with agrochemicals can lead to an increased resistance to pyrethroids in adults, and also that agrochemical formulated products not sold as insecticides can indeed kill insects. Resistance to deltamethrin increased over the generations in all selected lines (ins line, non-ins line, mix line). Such increased resistance was not associated with a significant increase of the L995F *kdr* mutation affecting the voltage-gated sodium channel targeted by pyrethroids [63–65]. However, one should note that this *kdr* mutation significantly decreased through generations in the non-selected line while its frequency remained stable in selected lines. Though drift effect may have occurred, this supports fitness costs associated with this mutation in absence of insecticide [66]. Hence, the stable frequency observed in all selected lines may indicate that insecticide and non-insecticide mixtures still exerted a moderate selection pressure on the VGSC. In *An. gambiae*, the L995F ‘*kdr*’ mutation has been widely observed in association with pyrethroid and DDT resistance throughout Africa [39, 63, 67]. In addition, a high frequency of this mutation is also frequently observed in intensive agricultural areas where crop protection strategies mainly rely on the use of chemical insecticides [18, 19, 41, 68]. The maintaining of the *kdr* L995F mutation in the non-insecticide line may be explained by the presence of an organochlorine (chlorothalonil, sold as a fungicide) in the non-insecticide agrochemical mixture which alike pyrethroids and DDT may exert a selection pressure on insect VGSC [69]. Altogether, the early rise of deltamethrin resistance in selected lines can hardly be explained by the presence of the L995F *kdr* mutation suggesting that other deltamethrin resistance alleles were selected by pesticide mixtures.

In contrast to deltamethrin and despite the presence of carbamates in both the insecticide and non-insecticide mixtures (carbofuran and carbendazime), no resistance of adults to bendiocarb was observed. This absence of bendiocarb resistance was associated with a slow decrease of the G119S ace1 mutation commonly associated to carbamate and organophosphate resistance in *An. gambiae* [17, 24, 25, 36, 70]. This trend might be explained by the low initial frequency of the ace1 mutation in the parental line and its significant fitness cost [17, 71]. This trend may also indicate a lower selection pressure exerted by carbamates and organophosphates present in the agrochemical mixtures as compared to other insecticides, such as pyrethroids and organochlorines. In accordance with this, the resistance of adult mosquitoes to bendiocarb and malathion is often less marked in agriculture intensive areas as compared to DDT and pyrethroids [18, 19]. In addition, it is likely that negative interactions occurred between the different chemicals present in the insecticide mixture, which might have decreased the selection pressure exerted by carbamates. In *An. gambiae*, negative interference between insecticides from different families has been shown with opposite effects on a key detoxification enzyme [56].

### Larval selection with agrochemical mixtures selects for a broad chemical stress response in adults

GO terms enrichment analysis showed a marked enrichment in molecular functions associated with xenobiotic detoxification or cuticle exoskeleton in all selected lines. This trend was also evident from the large overlap of over-transcribed candidate genes between the three selected lines. Such a broad adaptive response to different chemical mixtures was also supported by polymorphism data showing common selection signatures between the three selected lines and low genetic distances between them as inferred by PCA. It is very likely that the common variations observed in the selected lines are adaptative responses rather than caused by genetic drift events affecting solely the unselected line, because polymorphism rate was still higher in the control line than in selected lines. Altogether, both gene expression and polymorphism data support the selection of a broad and generalist response to chemical stress affecting multiple loci and acting on various traits such as xenobiotic penetration and metabolism.

Among phase I detoxification enzymes, several P450s were over-transcribed in one or multiple selected lines. These include key resistance genes like *CYP6P3* or *CYP6M2* whose role in pyrethroid resistance has been functionally or genetically validated [58, 72] together with other P450s (e.g. *CYP12F4*, *CYP6Z2* and *CYP9M1*) previously associated with resistance using laboratory or field approaches [73, 74]. The response of P450s to larval selection with agrochemical mixtures is further supported by the selection signature observed at the *CYP6M* resistance locus on Chromosome 3R which contains 14 CYP6 genes from the *CYP6Y*, *CYP6M* and *CYP6Z* subfamilies.

Among phase II enzymes (transferases), multiple GSTs and UDPGTs were over-transcribed or affected by selection signatures following selection while agrochemical mixtures. An over-transcription of *GSTe2* was detected upon selection with the insecticide mixture together with a positive selection signature at the GSTE locus. The role of *GSTe2* in DDT resistance has been demonstrated in *An. gambiae* and other mosquito species [35, 75, 76]. This gene and other epsilon GSTs have also been implicated in resistance to various insecticides including pyrethroids and organophosphates in mosquitoes [77] supporting their adaptive role toward various insecticides. Multiple UDPGTs were over-transcribed in one or multiple selected lines while others were associated with selection signatures. These phase II conjugating enzymes are thought to play a key role in xenobiotic detoxification pathways in most organisms [78–80]. In insects including mosquitoes, the association of UDPGTs and P450s in pyrethroid metabolism pathways has frequently been observed [75, 79, 81] but their role in the detoxification of other agrochemicals is likely. Other proteins likely contributing to xenobiotic metabolism were over-transcribed and/or affected by selection signatures in selected lines. This includes various phase I enzymes such as aldehyde oxidase or epoxide hydrolase [12, 82] but also multiple ABC transporters known to contribute to the excretion of xenobiotics and their conjugated metabolites [83, 84]. In mosquitoes, ABC transporters have been frequently associated with pyrethroid resistance though their complexity and late positioning in detoxification pathways makes their functional validation challenging [85, 86]. Among non-enzymatic binding proteins, sensory appendage proteins (SAP) appear as likely involved in the broad defence against xenobiotics, since a selection signature appears at the SAP locus in all selected lines. SAP proteins were shown to bind various xenobiotics, among which SAP2 was shown to confer pyrethroid resistance in *An. gambiae* [87]. Also, two Niemann Pick type C2 (NPC2) genes were strongly upregulated in the three selected lines. NPC proteins, initially identified as cholesterol-like transporters, have been suggested to bind xenobiotics and might therefore contribute to their sequestration [88, 89]. Interestingly, the neurodegenerative Niemann-Pick type C (NPC) disease caused by mutations in NPC genes was also associated with a defective P450-mediated drug metabolism in mouse supporting a cross talk with detoxification pathways [90]. Xenobiotic response has also been associated with a higher tolerance to oxidative stress in various insects including mosquitoes [91–93]. Such response was also apparent in the transcriptomic dataset with multiple red/ox enzymes (haem peroxidases, thioredoxin peroxidase, superoxide dismutase) being differentially transcribed in selected lines.

Finally, several structural cuticle protein genes were over-transcribed in the selected lines. Expression patterns were relatively conserved between lines, supporting the hypothesis of a generalist adaptation to chemical stress. Among over-transcribed genes were two members of the CPLCG gene cluster located on chromosome 3R, including CPLCG5 known to play a key role in pyrethroid resistance [34]. This gene cluster also shows a clear selection signature upon insecticide mixture selection. A cuticular component of xenobiotic resistance is further supported by the over-transcription of chitin synthase, an enzyme playing a key role in cuticle formation [94]. Deciphering whether the over-transcription of these multiple cuticle proteins is associated with physiological cuticle alteration (cuticle thickening and/or altered insecticide penetration) in selected lines deserves further work.

### Agrochemicals as a key selection pressure contributing to insecticide resistance in malaria vectors

Overall, the present work confirms that agrochemical mixtures contaminating mosquito breeding sites represent a significant selection pressure enhancing the ability of adult mosquitoes to resist vector control insecticides. The deltamethrin resistance phenotype observed upon selection with agrochemical mixtures was associated with a broad adaptative response to chemical stress involving detoxification- and cuticle-related pathways. Such multigenic adaptation to chemical stress was largely conserved among the different selection regimes suggesting that a large proportion of selected genes do not specifically respond to a particular agrochemical but were rather selected by multiple compounds.

One major difference between selection pressures represented by vector control versus agriculture stands on their specificity: chemical insecticides used for vector control can be considered as a specific selection pressure (limited number of active ingredients used at a time, most of the time as a single product) while chemical used in agriculture likely represent a broader selection pressure (higher diversity of active ingredients used sequentially or as mixtures). In addition, different agrochemicals (or their metabolites) may accumulate in mosquito breeding sites leading to the exposure of mosquito larvae to complex xenobiotic mixtures [40, 42]. In such situation, the higher complexity of agriculture-based selection pressures likely favours the selection of generalist resistance mechanisms (i.e. broad spectrum detoxification enzymes, sequestration proteins, cuticle modifications) as opposed to more specific resistance mechanisms (e.g. target-site mutations and a few detoxification enzymes) that are often selected by vector control interventions [38, 39, 44, 59]. Though no cross-resistance between deltamethrin and bendiocarb was observed in the selected lines, the diversity and generalist nature of the resistance alleles selected by agrochemical mixtures agrees with the multi-resistance phenotypes frequently observed in intense agriculture areas [45, 95].

## Conclusion

Altogether, the present study confirms that mosquitoes can cope with the variety of anthropogenic xenobiotics encountered in their larval environment through multi-genic adaptive trajectories which in turn may impact various adult traits including resistance to vector control insecticides. Given the limited number of active ingredients available for public health, this may have a significant impact on the management of resistance in malaria vectors and calls for an integrated management of resistance between agriculture and vector control. Whether other key vector biological functions (e.g. reproduction, development, aging, behaviour) are impacted by agrochemicals may deserve further attention as this may affects the global ecology of vectors and malaria transmission though Africa.

### Supplementary Information

**Additional file 1: Figure S1.** Venn diagrams. Venn diagrams showing the numbers of genes significantly differentially transcribed in each selected line as compared to the control line (FC ≥ 1.5-fold in either direction and corrected P value ≤ 0.005). The number of resistance candidate genes are shown within brackets.

**Additional file 2: Figure S2.** GO terms enrichment analysis. Enrichment analyses were performed for each line using genes significantly over- and under-transcribed separately (test lists) as compared to all genes detected by RNA-seq (reference list). Enrichment analyses were performed with the functional annotation tool DAVID (http://david.abcc.ncifcrf.gov) on all terms belonging to the ‘molecular function’ GO family. Only genes showing a corrected Fisher’s test P value ≤ 0.05 and represented by at least 4 genes are shown.

**Additional file 3: Figure S3.** Principal Component Analysis of polymorphism data. PCA was performed using the frequency of all bi-allelic SNPs identified in each replicate of all lines as compared to the reference genome. Only the three first PCA axes are shown, accounting for 96.3% of the total variance.

**Additional file 4: Table S4.** Transcription data obtained for all genes detected by RNA-seq.

**Additional file 5: Table S5.** Gene-level polymorphism data obtained from RNA-seq.

## Data Availability

RNA-seq sequence data reported in this study have been deposited to the European Nucleotide Archive (ENA; http://www.ebi.ac.uk/ena) under the accession numbers E-MTAB-12756.

## Abbreviations

WHO: World Health Organization
IRS: Indoor residual spraying
ITN: Long-lasting insecticidal nets
VGSC: Voltage-gated sodium channel
kdr: Knock-down resistance
ace1: Acetylcholinesterases1

## References

[1] Sinka ME, Bangs MJ, Manguin S, Rubio-Palis Y, Chareonviriyaphap T, Coetzee M (2012). A global map of dominant malaria vectors. Parasit Vectors.

[2] Wolie RZ, Koffi AA, Ahoua Alou LP, Sternberg ED, N’Nan-Alla O, Dahounto A (2021). Evaluation of the interaction between insecticide resistance-associated genes and malaria transmission in *Anopheles gambiae sensu lato* in central Côte d’Ivoire. Parasit Vectors.

[3] WHO (2022). World malaria report 2022.

[4] Wilson AL, Courtenay O, Kelly-Hope LA, Scott TW, Takken W, Torr SJ (2020). The importance of vector control for the control and elimination of vector-borne diseases. PLoS Negl Trop Dis.

[5] van den Berg H, Velayudhan R, Yadav RS (2021). Management of insecticides for use in disease vector control: lessons from six countries in Asia and the Middle East. PLoS Negl Trop Dis.

[6] WHO. World malaria report 2019. Geneva: World Health Organization; 2019. https://www.who.int/news-room/fact-sheets/detail/malaria

[7] Guillet P, Chandre F, Mouchet J (1997). L’utilisation des insecticides en santé publique: état et perspectives. Med Mal Infect.

[8] WHO (2015). Global malaria programme. Eliminating malaria.

[9] WHO (2020). World malaria report 2020.

[10] Hemingway J, Ranson H (2000). Insecticide resistance in insect vectors of human disease. Annu Rev Entomol.

[11] Hemingway J, Hawkes NJ, McCarroll L, Ranson H (2004). The molecular basis of insecticide resistance in mosquitoes. Insect Biochem Mol Biol.

[12] Hemingway J, Coleman M, Paton M, McCarroll L, Vaughan A, DeSilva D (2000). Aldehyde oxidase is coamplified with the World’s most common *Culex* mosquito insecticide resistance-associated esterases. Insect Mol Biol.

[13] Martinez-Torres D, Chandre F, Williamson MS, Darriet F, Bergé JB, Devonshire AL (1998). Molecular characterization of pyrethroid knockdown resistance (kdr) in the major malaria vector *Anopheles gambiae* s.s.. Insect Mol Biol.

[14] Silva APB, Santos JMM, Martins AJ (2014). Mutations in the voltage-gated sodium channel gene of anophelines and their association with resistance to pyrethroids—a review. Parasit Vectors.

[15] Berthomieu A, Marquine M, Raymond M (2004). The unique mutation in ace-1 giving high insecticide resistance is easily detectable in mosquito vectors. Insect Mol Biol.

[16] Bourguet D, Pasteur N, Bisset J, Raymond M (1996). Determination of ace.1 genotypes in single mosquitoes: toward an ecumenical biochemical test. Pestic Biochem Physiol.

[17] Assogba BS, Djogbénou LS, Milesi P, Berthomieu A, Perez J, Ayala D (2015). An ace-1 gene duplication resorbs the fitness cost associated with resistance in *Anopheles gambiae*, the main malaria mosquito. Sci Rep.

[18] Camara S, Koffi AA, Ahoua Alou LP, Koffi K, Kabran JPK, Koné A (2018). Mapping insecticide resistance in *Anopheles gambiae* (s.l.) from Côte d’Ivoire. Parasit Vectors.

[19] Fodjo BK, Koudou BG, Tia E, Saric J, N’dri PB, Zoh MG (2018). Insecticides resistance status of *An. gambiae* in areas of varying agrochemical use in Côte D’Ivoire. BioMed Res Int.

[20] Miles A, Harding NJ, Bottà G, Clarkson CS, Antão T, Kozak K (2017). Genetic diversity of the African malaria vector *Anopheles gambiae*. Nature.

[21] Chouaïbou M, Kouadio FB, Tia E, Djogbenou L (2017). First report of the East African kdr mutation in an *Anopheles gambiae* mosquito in Côte d’Ivoire. Wellcome Open Research.

[22] Verhaeghen K, Van Bortel W, Roelants P, Backeljau T, Coosemans M (2006). Detection of the East and West African kdr mutation in *Anopheles gambiae* and *Anopheles arabiens*is from Uganda using a new assay based on FRET/Melt Curve analysis. Malar J.

[23] Dabiré RK, Namountougou M, Diabaté A, Soma DD, Bado J, Toé HK (2014). Distribution and frequency of kdr mutations within *Anopheles gambiae* s.l. populations and first report of the Ace1G119S mutation in *Anopheles arabiensis* from Burkina Faso (West Africa). PLoS ONE.

[24] Keïta M, Sogoba N, Kané F, Traoré B, Zeukeng F, Coulibaly B (2021). Multiple resistance mechanisms to pyrethroids insecticides in *Anopheles gambiae sensu lato* population from Mali, West Africa. Africa J Infect Dis.

[25] Elanga-Ndille E, Nouage L, Ndo C, Binyang A, Assatse T, Nguiffo-Nguete D (2019). The g119s acetylcholinesterase (Ace-1) target site mutation confers carbamate resistance in the major malaria vector *Anopheles gambiae* from Cameroon: a challenge for the coming irs implementation. Genes (Basel).

[26] Essandoh J, Yawson AE, Weetman D (2013). Acetylcholinesterase (Ace-1) target site mutation 119S is strongly diagnostic of carbamate and organophosphate resistance in *Anopheles gambiae* s.s. and *Anopheles coluzzii* across southern Ghana. Malar J.

[27] Osta MA, Rizk ZJ, Labbé P, Weill M, Knio K (2012). Insecticide resistance to organophosphates in *Culex pipiens* complex from Lebanon. Parasit Vectors.

[28] Zoh DD, Ahoua Alou LP, Toure M, Pennetier C, Camara S, Traore DF (2018). The current insecticide resistance status of *Anopheles gambiae* (s.l.) (Culicidae) in rural and urban areas of Bouaké, Côte d’Ivoire. Parasit Vectors.

[29] Vontas J, Katsavou E, Mavridis K (2020). Cytochrome P450-based metabolic insecticide resistance in *Anopheles* and *Aedes* mosquito vectors: muddying the waters. Pestic Biochem Physiol.

[30] Strode C, Wondji CS, David JP, Hawkes NJ, Lumjuan N, Nelson DR (2008). Genomic analysis of detoxification genes in the mosquito *Aedes aegypti*. Insect Biochem Mol Biol.

[31] Ranson H, Guessan RN, Lines J, Moiroux N, Nkuni Z, Corbel V (2010). Pyrethroid resistance in African anopheline mosquitoes: what are the implications for malaria control ?. Trends Parasitol.

[32] Vannini L, Reed TW, Willis JH (2014). Temporal and spatial expression of cuticular proteins of *Anopheles gambiae* implicated in insecticide resistance or differentiation of M/S incipient species. Parasit Vectors.

[33] Yahouédo GA, Chandre F, Rossignol M, Ginibre C, Balabanidou V, Mendez NGA (2017). Contributions of cuticle permeability and enzyme detoxification to pyrethroid resistance in the major malaria vector *Anopheles gambiae*. Sci Rep.

[34] Huang Y, Guo Q, Sun X, Zhang C, Xu N, Xu Y (2018). Culex pipiens pallens cuticular protein CPLCG5 participates in pyrethroid resistance by forming a rigid matrix. Parasit Vectors.

[35] Riveron JM, Yunta C, Ibrahim SS, Djouaka R, Irving H, Menze BD (2014). A single mutation in the GSTe2 gene allows tracking of metabolically based insecticide resistance in a major malaria vector. Genome Biol.

[36] Ranson H, Edi CVA, Koudou BG, Jones CM, Weetman D (2012). Multiple-insecticide resistance in *Anopheles gambiae* mosquitoes, Southern Côte d’Ivoire. Emerg Infect Dis.

[37] Chandor-Proust A, Bibby J, Régent-Kloeckner M, Roux J, Guittard-Crilat E, Poupardin R (2013). The central role of mosquito cytochrome P450 CYP6Zs in insecticide detoxification revealed by functional expression and structural modelling. Biochem J.

[38] Czeher C, Labbo R, Arzika I, Duchemin J (2008). Evidence of increasing Leu-Phe knockdown resistance mutation in *Anopheles gambiae* from Niger following a nationwide long-lasting insecticide-treated nets implementation. Malar J.

[39] Aïzoun N, Aïkpon R, Akogbéto M (2014). Evidence of increasing L1014F kdr mutation frequency in *Anopheles gambiae* s.l. pyrethroid resistant following a nationwide distribution of LLINs by the Beninese National Malaria Control Programme. Asian Pac J Trop Biomed.

[40] Chouaïbou MS, Fodjo BK, Fokou G, Allassane OF, Koudou BG, David JP (2016). Influence of the agrochemicals used for rice and vegetable cultivation on insecticide resistance in malaria vectors in southern Côte d’Ivoire. Malar J.

[41] Hien AS, Soma DD, Hema O, Bayili B, Namountougou M, Gnankiné O (2017). Evidence that agricultural use of pesticides selects pyrethroid resistance within *Anopheles gambiae* s.l. populations from cotton growing areas in Burkina Faso, West Africa. PLoS ONE.

[42] Yadouleton A, Martin T, Padonou G, Chandre F, Asidi A, Djogbenou L (2011). Cotton pest management practices and the selection of pyrethroid resistance in *Anopheles gambiae* population in Northern Benin. Parasit Vectors.

[43] Hawkins NJ, Bass C, Dixon A, Neve P (2019). The evolutionary origins of pesticide resistance. Biol Rev Camb Philos Soc.

[44] Nkya TE, Poupardin R, Laporte F, Akhouayri I, Mosha F, Magesa S (2014). Impact of agriculture on the selection of insecticide resistance in the malaria vector *Anopheles gambiae:* a multigenerational study in controlled conditions. Parasit Vectors.

[45] Oumbouke WA, Pignatelli P, Barreaux AMG, Tia IZ, Koffi AA, Ahoua Alou LP (2020). Fine scale spatial investigation of multiple insecticide resistance and underlying target-site and metabolic mechanisms in *Anopheles gambiae* in central Côte d’Ivoire. Sci Rep.

[46] WHO. Test procedures for insecticide resistance monitoring in malaria vector mosquitoes, 2nd edn; 2016. https://fctc.who.int/publications/i/item/9789241511575

[47] Abbott WS (1987). A method of computing the effectiveness of an insecticide. J Am Mosq Control Assoc.

[48] Khumallambam D, Kshetrimayum P, Nandeibam S, Huidrom S (2013). An efficient protocol for total DNA extraction from the members of order Zingiberales- suitable for diverse PCR based downstream applications. Springerplus.

[49] Nadler SG, Tritschler D, Haffar OK, Blake J, Bruce AG, Cleaveland JS (1997). Differential expression and sequence-specific interaction of karyopherin α with nuclear localization sequences. J Biol Chem.

[50] Benjamini Y, Hochberg Y (1995). Controlling the false discovery rate: a practical and powerful approach to multiple testing. J R Stat Soc Ser B (Methodological).

[51] Huang DW, Sherman BT, Lempicki RA (2009). Bioinformatics enrichment tools: Paths toward the comprehensive functional analysis of large gene lists. Nucleic Acids Res.

[52] Saeed AI, Sharov V, White J, Li J, Liang W, Bhagabati N (2003). TM4: A free, open-source system for microarray data management and analysis. Biotechniques.

[53] Pearson KLIII (1901). On lines and planes of closest fit to systems of points in space. The London, Edinburgh, and Dublin Philos Magaz J Sci.

[54] Dray S, Dufour AB (2007). The ade4 package: implementing the duality diagram for ecologists. J Stat Softw.

[55] Foll M, Gaggiotti O (2008). A genome-scan method to identify selected loci appropriate for both dominant and codominant markers: a Bayesian perspective. Genetics.

[56] Adolfi A, Poulton B, Anthousi A, Macilwee S, Ranson H, Lycett GJ (2019). Functional genetic validation of key genes conferring insecticide resistance in the major African malaria vector, *Anopheles gambiae*. Proc Natl Acad Sci USA.

[57] Müller P, Warr E, Stevenson BJ, Pignatelli PM, Morgan JC, Steven A (2008). Field-caught permethrin-resistant *Anopheles gambiae* overexpress CYP6P3, a P450 that metabolises pyrethroids. PLoS Genet.

[58] Wagah MG, Korlević P, Clarkson C, Miles A, Lawniczak MKN, Makunin A (2021). Genetic variation at the Cyp6m2 putative insecticide resistance locus in *Anopheles gambiae* and *Anopheles coluzzii*. Malar J.

[59] Zoh MG, Tutagata J, Fodjo BK, Mouhamadou CS, Sadia CG, McBeath J (2022). Exposure of *Anopheles gambiae* larvae to a sub-lethal dose of an agrochemical mixture induces tolerance to adulticides used in vector control management. Aquat Toxicol.

[60] Wang Y, Cheng P, Jiao B, Song X, Wang H, Wang H (2020). Investigation of mosquito larval habitats and insecticide resistance in an area with a high incidence of mosquito-borne diseases in Jining, Shandong Province. PLoS ONE.

[61] Poupardin R, Riaz MA, Vontas J, David JP, Reynaud S (2010). Transcription profiling of eleven cytochrome p450s potentially involved in xenobiotic metabolism in the mosquito *Aedes aegypti*. Insect Mol Biol.

[62] Bara J, Montgomery A, Muturi J (2014). Sublethal effects of atrazine and glyphosate on life history traits of *Aedes aegypti* and *Aedes albopictus* (Diptera: Culicidae). Parasitol Res.

[63] Diallo B, Lee Y, Reimer L, Fondjo E, Patchoke S, Ng A (2008). Relationship between kdr mutation and resistance to pyrethroid and DDT insecticides in natural populations of *Anopheles gambiae*. J Med Entomol.

[64] Perera YC, Garcia GP, Segura KV, Monroy BL, Sanchez IPR, Lenhart A (2020). Impact of deltamethrin selection on kdr mutations and insecticide detoxifying enzymes in *Aedes aegypti* from Mexico. Parasit Vectors.

[65] Auteri M, La Russa F, Blanda V, Torina A. Insecticide resistance associated with kdr mutations in *Aedes albopictus*:. Biomed Res Int. 2018;2018:3098575. an update on worldwide evidences 10.1155/2018/3098575 PMC6098900 30175124

[66] Amer K, Saavedra-Rodriguez K, Black WC, Gray EM (2021). Effect of selection for pyrethroid resistance on abiotic stress tolerance in Aedes aegypti from Merida, Yucatan, Mexico. Insects.

[67] Lynd A, Oruni A, Van’T Hof AE, Morgan JC, Naego LB, Pipini D (2018). Insecticide resistance in *Anopheles gambiae* from the northern Democratic Republic of Congo, with extreme knockdown resistance (kdr) mutation frequencies revealed by a new diagnostic assay. Malar J.

[68] Bamou R, Sonhafouo-Chiana N, Mavridis K, Tchuinkam T, Wondji CS, Vontas J (2019). Status of insecticide resistance and its mechanisms in *Anopheles gambiae* and *Anopheles coluzzii* populations from forest settings in South Cameroon. Genes (Basel).

[69] Narahashi T (1996). Neuronal ion channels as the target sites. Pharmacol Toxicol.

[70] Edi CV, Djogbénou L, Jenkins AM, Regna K, Muskavitch MAT, Poupardin R (2014). CYP6 P450 enzymes and ACE-1 duplication produce extreme and multiple insecticide resistance in the malaria mosquito *Anopheles gambiae*. PLoS Genet.

[71] Djogbénou L, Noel V, Agnew P (2010). Costs of insensitive acetylcholinesterase insecticide resistance for the malaria vector *Anopheles gambiae* homozygous for the G119S mutation. Malar J.

[72] Djouaka RF, Bakare AA, Coulibaly ON, Akogbeto MC, Ranson H, Hemingway J (2008). Expression of the cytochrome P450s, CYP6P3 and CYP6M2 are significantly elevated in multiple pyrethroid resistant populations of *Anopheles gambiae* s.s. from Southern Benin and Nigeria. BMC Genomics.

[73] Nardini L, Christian RN, Coetzer N, Koekemoer LL (2013). DDT and pyrethroid resistance in *Anopheles arabiensis* from South Africa. Parasit Vectors.

[74] Toé KH, N’Falé S, Dabiré RK, Ranson H, Jones CM (2015). The recent escalation in strength of pyrethroid resistance in *Anopheles coluzzi* in West Africa is linked to increased expression of multiple gene families. BMC Genomics.

[75] Lumjuan N, McCarroll L, Prapanthadara LA, Hemingway J, Ranson H (2005). Elevated activity of an Epsilon class glutathione transferase confers DDT resistance in the dengue vector, *Aedes aegypti* White star. Insect Biochem Mol Biol.

[76] Lumjuan N, Rajatileka S, Changsom D, Wicheer J, Leelapat P, La-aied Prapanthadara L (2011). The role of the *Aedes aegypti* Epsilon glutathione transferases in conferring resistance to DDT and pyrethroid insecticides. Insect Biochem Mol Biol.

[77] Atoyebi SM, Tchigossou GM, Akoton R, Riveron JM, Irving H, Weedall G (2020). Investigating the molecular basis of multiple insecticide resistance in a major malaria vector *Anopheles funestus* (sensu stricto) from Akaka-Remo, Ogun State, Nigeria. Parasit Vectors.

[78] Zhou Y, Fu WB, Si FL, Yan ZT, Zhang YJ, He QY (2019). UDP-glycosyltransferase genes and their association and mutations associated with pyrethroid resistance in *Anopheles sinensis* (Diptera: Culicidae). Malar J.

[79] Antonio-Nkondjio C, Poupardin R, Tene BF, Kopya E, Costantini C, Awono-Ambene P (2016). Investigation of mechanisms of bendiocarb resistance in *Anopheles gambiae* populations from the city of Yaoundé, Cameroon. Malar J.

[80] Helvecio E, Romão TP, de Carvalho-Leandro D, de Oliveira IF, Cavalcanti AEHD, Reimer L (2020). Polymorphisms in GSTE2 is associated with temephos resistance in *Aedes aegypti*. Pestic Biochem Physiol.

[81] Kaplanoglu E, Chapman P, Scott IM, Donly C (2017). Overexpression of a cytochrome P450 and a UDP-glycosyltransferase is associated with imidacloprid resistance in the Colorado potato beetle, *Leptinotarsa decemlineata*. Sci Rep.

[82] Coleman M, Vontas JG, Hemingway J (2002). Molecular characterization of the amplified aldehyde oxidase from insecticide resistant *Culex quinquefasciatus*. Eur J Biochem.

[83] Wu C, Chakrabarty S, Jin M, Liu K, Xiao Y (2019). Insect ATP-binding cassette (ABC) transporters: roles in xenobiotic detoxification and Bt insecticidal activity. Int J Mol Sci.

[84] Chen X-Y, Yang Y, Wang J-Q, Wu Z-X, Li J, Chen Z-S (2021). Overexpression of ABCC1 confers drug resistance to betulin. Front Oncol.

[85] Pignatelli P, Ingham VA, Balabanidou V, Vontas J, Lycett G, Ranson H (2018). The *Anopheles gambiae* ATP-binding cassette transporter family: phylogenetic analysis and tissue localization provide clues on function and role in insecticide resistance. Insect Mol Biol.

[86] Epis S, Porretta D, Mastrantonio V, Comandatore F, Sassera D, Rossi P (2014). ABC transporters are involved in defense against permethrin insecticide in the malaria vector *Anopheles stephensi*. Parasit Vectors.

[87] Ingham VA, Anthousi A, Douris V, Harding NJ, Lycett G, Morris M (2020). A sensory appendage protein protects malaria vectors from pyrethroids. Nature.

[88] Gong Y, Duvvuri M, Duncan MB, Liu J, Krise JP (2006). Niemann-Pick C1 protein facilitates the efflux of the anticancer drug daunorubicin from cells according to a novel vesicle-mediated pathway. J Pharmacol Exp Ther.

[89] Naren D, Wu J, Gong Y, Yan T, Wang K, Xu W (2016). Niemann-Pick disease type C1(NPC1) is involved in resistance against imatinib in the imatinib-resistant Ph+ acute lymphoblastic leukemia cell line SUP-B15/RI. Leuk Res.

[90] Nicoli ER, Eisa NA, Cluzeau CVM, Wassif CA, Gray J, Burkert KR (2016). Defective cytochrome p450-catalysed drug metabolism in Niemann-Pick type C disease. PLoS ONE.

[91] Abdollahi M, Ranjbar A, Shadnia S, Nikfar S, Rezaie A (2004). Pesticides and oxidative stress: a review. Med Sci Monit.

[92] Champion CJ, Xu J (2018). Redox state affects fecundity and insecticide susceptibility in *Anopheles gambiae*. Sci Rep.

[93] Oliver SV, Brooke BD (2016). The role of oxidative stress in the longevity and insecticide resistance phenotype of the major malaria vectors *Anopheles arabiensis* and *Anopheles funestus*. PLoS ONE.

[94] Yang X, Xu Y, Yin Q, Zhang H, Yin H, Sun Y (2021). Physiological characterization of chitin synthase A responsible for the biosynthesis of cuticle chitin in *Culex pipiens pallens* (Diptera: Culicidae). Parasit Vectors.

[95] Wipf NC, Duchemin W, Kouadio FPA, Fodjo BK, Sadia CG, Mouhamadou CS (2022). Multi-insecticide resistant malaria vectors in the field remain susceptible to malathion, despite the presence of Ace1 point mutations. PLoS Genet.

