# Supplementary figures and images for: The impact of agrochemical pollutant mixtures on the selection of insecticide resistance in the malaria vector Anopheles gambiae: insights from experimental evolution and transcriptomics
Source: Malar J. 2024 Mar 5;23:69. doi: 10.1186/s12936-023-04791-0 (PMC10916200; doi:10.1186/s12936-023-04791-0)

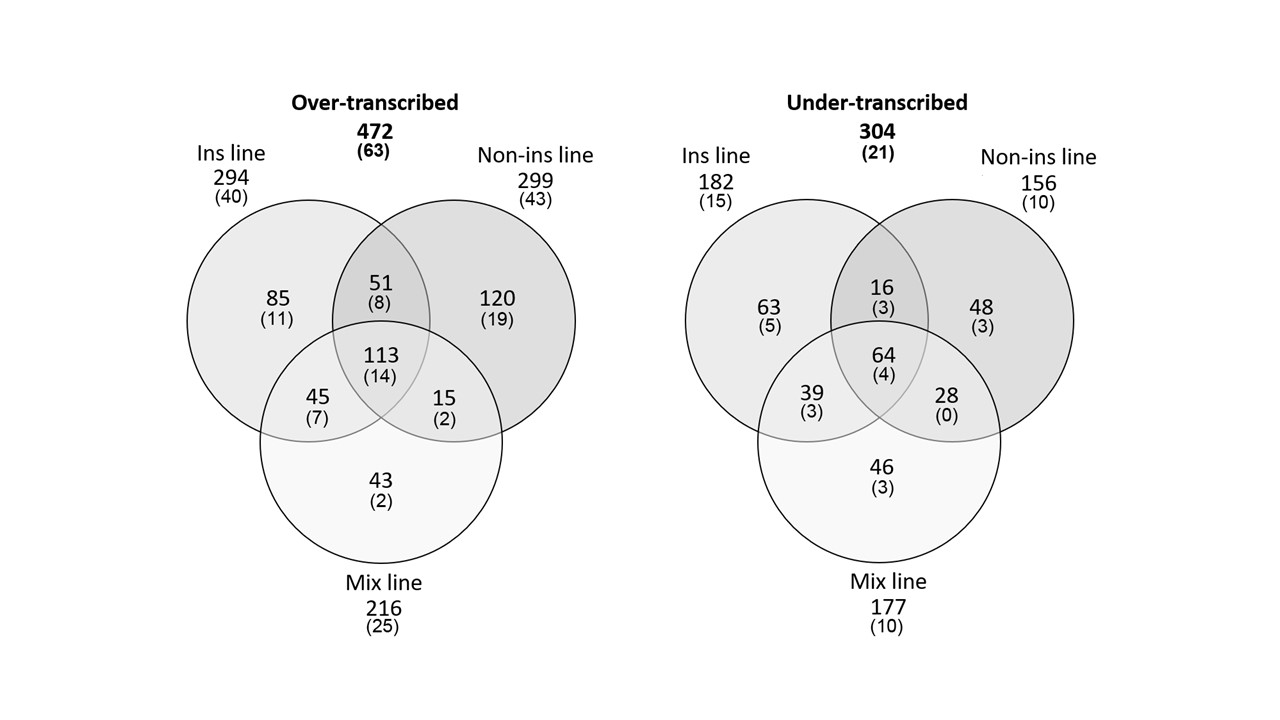

Supplement: Supplementary file 1 — Additional file 1: Figure S1. Venn diagrams. Venn diagrams showing the numbers of genes significantly differentially transcribed in each selected line as compared to the control line (FC ≥ 1.5-fold in either direction and corrected P value ≤ 0.005). The number of resistance candidate genes are shown within brackets. [file 12936_2023_4791_MOESM1_ESM.jpg]

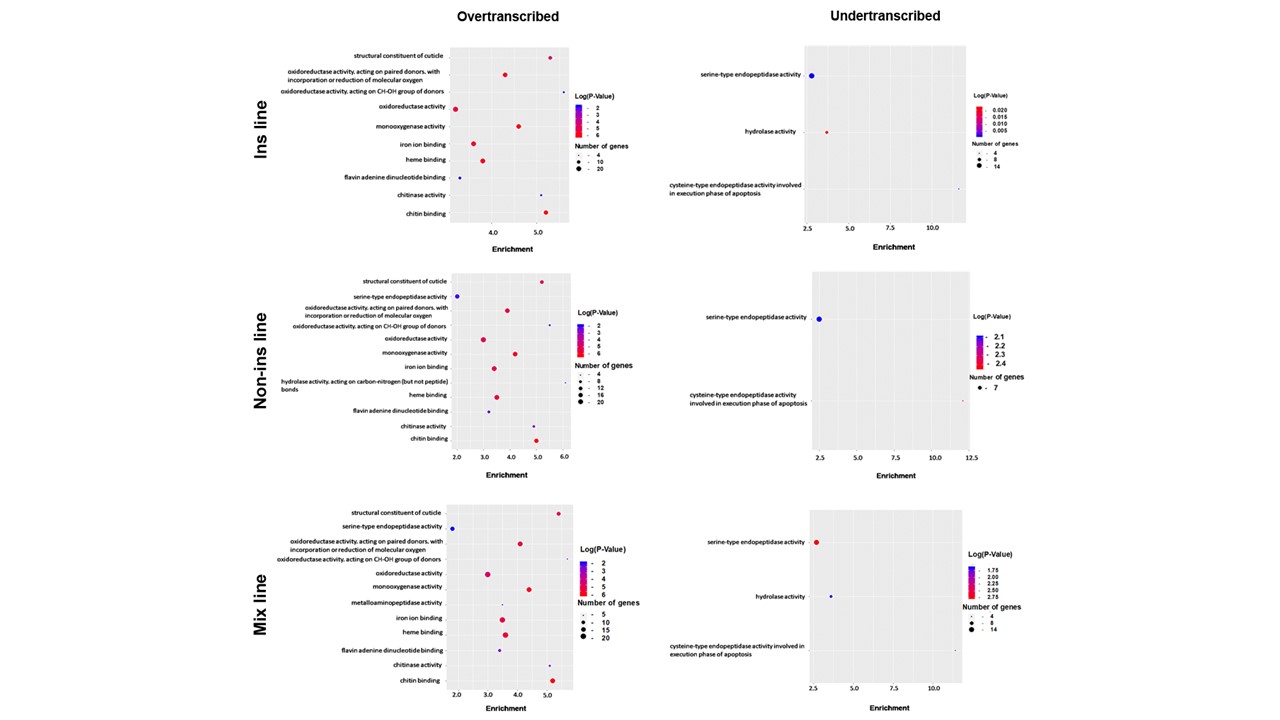

Supplement: Supplementary file 2 — Additional file 2: Figure S2. GO terms enrichment analysis. Enrichment analyses were performed for each line using genes significantly over- and under-transcribed separately (test lists) as compared to all genes detected by RNA-seq (reference list). Enrichment analyses were performed with the functional annotation tool DAVID (http://david.abcc.ncifcrf.gov) on all terms belonging to the ‘molecular function’ GO family. Only genes showing a corrected Fisher’s test P value ≤ 0.05 and represented by at least 4 genes are shown. [file 12936_2023_4791_MOESM2_ESM.jpg]

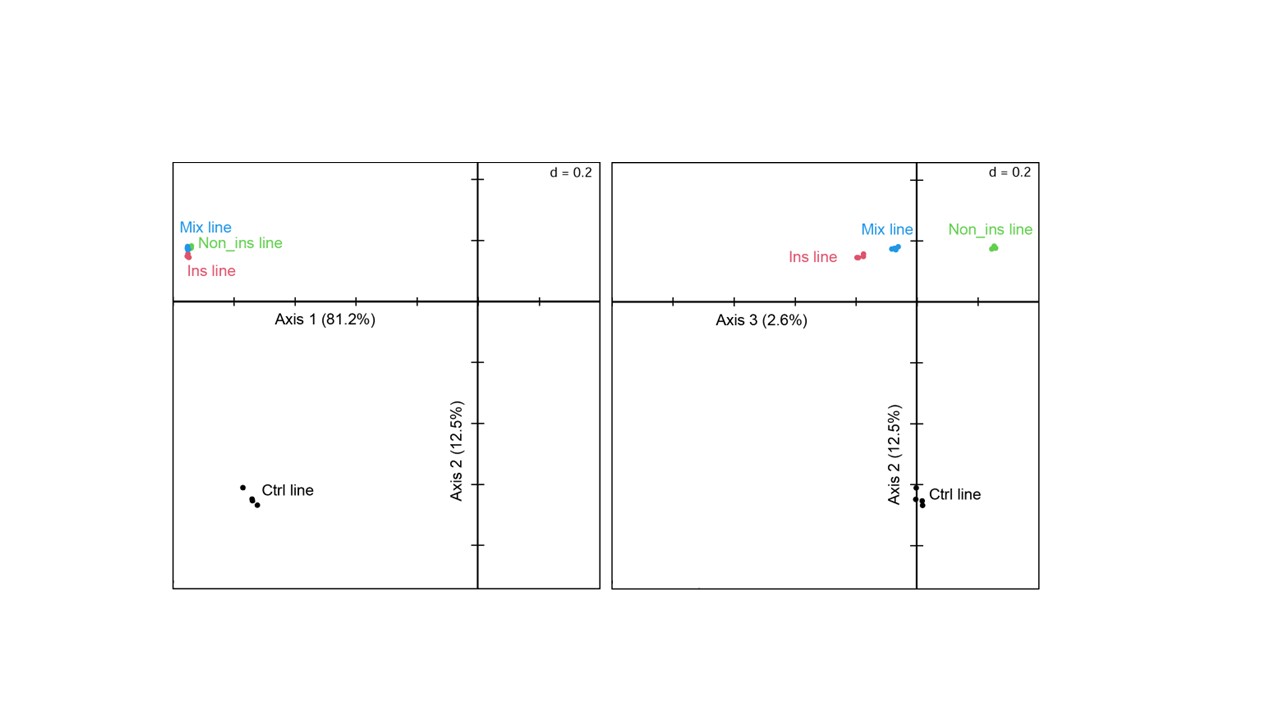

Supplement: Supplementary file 3 — Additional file 3: Figure S3. Principal Component Analysis of polymorphism data. PCA was performed using the frequency of all bi-allelic SNPs identified in each replicate of all lines as compared to the reference genome. Only the three first PCA axes are shown, accounting for 96.3% of the total variance. [file 12936_2023_4791_MOESM3_ESM.jpg]
